# Myocardial Infarction With Non-obstructive Coronary Arteries (MINOCA): A Clinical Conundrum

**DOI:** 10.7759/cureus.32108

**Published:** 2022-12-01

**Authors:** Endurance O Evbayekha, Okelue E Okobi, Oyintoun-emi Ozobokeme, Abosede Okoduwa, Quinn K Simbeye, Emmanuel Egberuare, Mercy O Koroyin, Ebikiye G Angaye, Ijeoma C Izundu, Mofeyisade E Okunlola

**Affiliations:** 1 Internal Medicine, St. Luke's Hospital, St. Louis, USA; 2 Family Medicine, Arizona State University, Tempe, USA; 3 Family Medicine, Lakeside Medical Center, Belle Glade, USA; 4 Medicine, Central Michigan University College of Medicine, Mount Pleasant, USA; 5 Pediatrics and Child Health, University of Manitoba, Winnipeg, CAN; 6 College of Medicine, University of Benin, Benin City, NGA; 7 Family Medicine, Springs Family Medical Clinic, Red Deer, CAN; 8 Urology, Southern Alberta Institute of Urology, Calgary, CAN; 9 Psychiatry, Priory Hospital Hayes Grove, Bromley, GBR; 10 Family Medicine, Diete Koki Memorial Hospital, Yenagoa, NGA; 11 Clinical Research, Pre/Post-Anesthetic Care Unit, Markham Stouffville Hospital, Markham, CAN; 12 Neurology, BODMED Neurological Centre, Ibadan, NGA; 13 Family and Community Medicine, Federal Medical Centre Owo, Owo, NGA

**Keywords:** non-obstructive coronary arteries, myocardial infarction type 1, cardiovascular ischemia, heart disease, coronary artery disease, myocardial infarction with non-obstructive coronary arteries (minoca)

## Abstract

Myocardial infarction (MI) is usually discussed in light of some occlusion to the coronary circulation. It usually occurs in the setting of well-established risk factors such as hypertension, obesity, coronary atherosclerosis, smoking, and male gender. However, a subset of this population does not follow the clinical presentation seen in traditional MI.

We present a case of acute MI in a middle-aged female with non-obstructive coronary arteries on coronary angiography.

## Introduction

Myocardial infarction with non-obstructive coronary arteries (MINOCA) can be described as having the symptomatology of acute myocardial infarction (MI), but still maintaining anatomically normal or near-normal coronary arteries, as evidenced by confirmation of a less than 50% occlusion on angiography [1]. A population-based study conducted in Alberta, Canada, revealed that of about 36,000 patients admitted for acute MI, 2,092 (5.8%) had MINOCA [2]. They also found the in-hospital mortality rate to be about 0.8% and the follow-up one-year mortality rate to be 5.3% [2]. Reportedly, MINOCA has a female predilection [3].

There is a variety of etiology of MINOCA. The following is a summary of the possible etiologies: Takotsubo cardiomyopathy, coronary artery spasm, coronary microvascular dysfunction, acute thrombosis, viral myocarditis, spontaneous coronary artery dissection, and coronary artery embolism [2].

This article presents a case of acute onset MI, with the workup eventually revealing non-obstructive coronary arteries.

## Case presentation

The patient was a 53-year-old Caucasian lady, a non-smoker with a past medical history consistent with hypertension (HTN), type 2 diabetes (T2DM), and severe obstructive sleep apnea (SOSA), requiring the use of a continuous positive airway pressure (CPAP) device.

**Figure 1:**
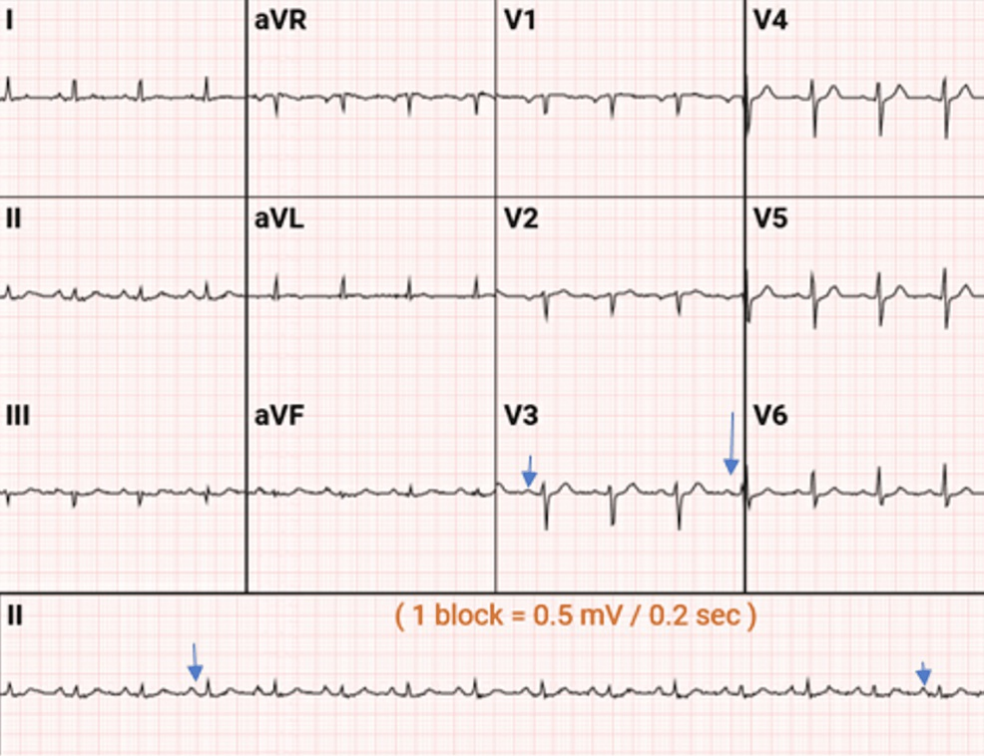
EKG was suspicious for atrial enlargement, as noted by the blue arrows. However, the consensus by the cardiologist was that the EKG was normal, with no evidence of acute ischemia

She was in her usual state of health until a few hours before the presentation. She was seated in her office when she started experiencing sudden onset, crushing anterior chest pain. She described the pain as sharp and crushing, substernal, constant, 9-10 out of 10 in intensity, and radiating to her throat. She reported that morphine and sublingual nitroglycerin relieved the pain, albeit partially. Furthermore, there was associated lower limb weakness and shortness of breath but no identifiable aggravating factors. A stat electrocardiogram (EKG) was obtained and was only suspicious for atrial enlargement but was otherwise within the normal range (Figure 1). Another EKG was obtained 48 hours later.

In the emergency department (ED), her vitals, including oxygen saturation, were all within normal limits. A computed tomography pulmonary angiography (CTPE) was negative for pulmonary embolism (PE). Her initial troponin was 4.3 ng/dL, and then it trended up to 6.36 ng/dL one hour later (normal is <0.03 ng/dL), and erythrocyte sedimentary rate (ESR) and C-reactive protein (CRP) were 57 mm/hour and 43.9, respectively. The acute coronary syndrome (ACS) protocol was initiated; the patient was started on nitroglycerin and heparin drip. Transesophageal echocardiography (TEE) showed a left ventricular ejection fraction (LVEF) of 45-50%. The patient was taken to the catheterization laboratory, and her coronary angiogram revealed an absence of obstructive coronary disease. Given this picture, the patient was worked-up to rule out myocarditis or other forms of cardiomyopathy. She underwent cardiac magnetic resonance imaging (MRI).

Echocardiogram

On echocardiogram, there was, however, a concomitant regional motion abnormality involving the left ventricular segment, with overall preservation of LVEF (54%) and right ventricular ejection fraction (RVEF) (51%) and normal right and left ventricular chamber size, as seen in Video 1 below.

**Video 1:** Echocardiogram showing heart chamber dynamics Interpretation: (1) Normal left ventricular cavity size. Normal left ventricular systolic function. The left ventricular ejection fraction is measured at 59%. (2) Normal right ventricular size. Normal right ventricular systolic function. (3) Mildly thickened pericardium with trace pericardial effusion.

Cardiac MRI

**Figure 2:**
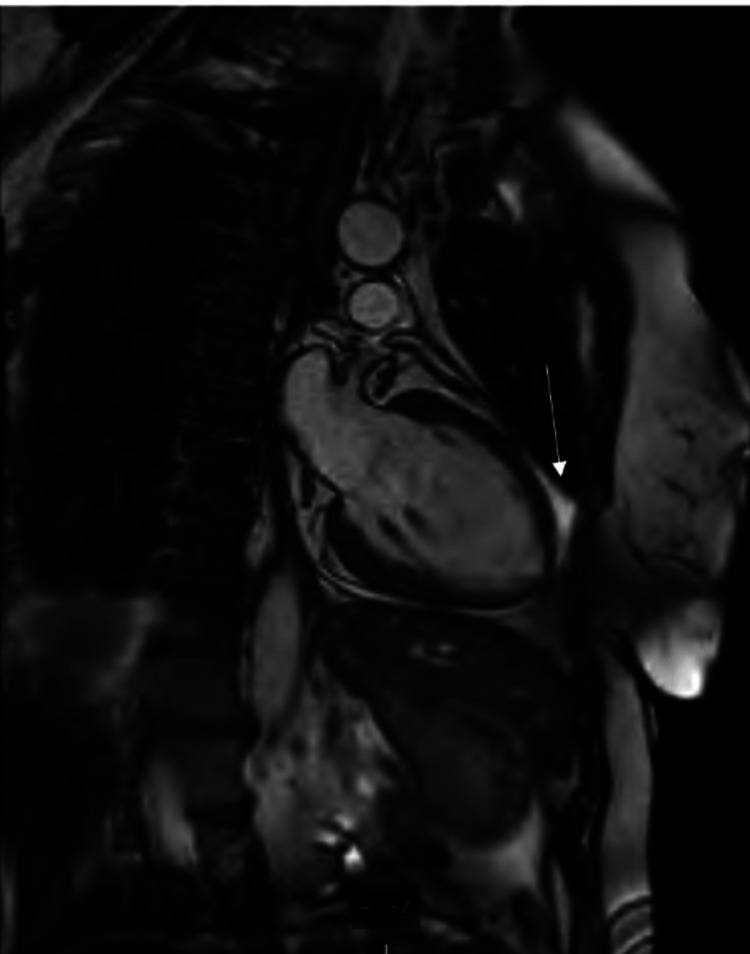
Transmural late gadolinium enhancement involving the isolated mid-anterolateral segment

The cardiac MRI revealed evidence of acute MI involving the mid-anterolateral segment, as suggested by transmural myocardial edema in Figure 2, and focal almost-transmural late gadolinium-enhancement (Figure 2) with focal areas of microvascular obstruction within dense necrotic regions of MI.

**Figure 3:**
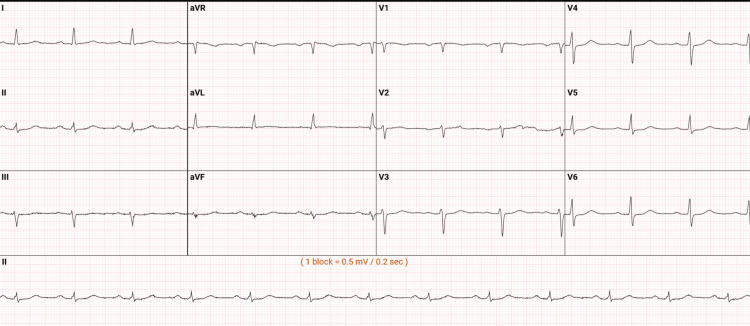
Repeat EKG 48 hours later without any significant change from the baseline presentation

Figure 3 shows a repeat EKG done 48 hours after the initial EKG. There were no significant changes in interpretation.

She was admitted to the telemetry floor, her home medications were resumed, the chest pain resolved over the next few hours, and her hospitalization course was uneventful. She was discharged five days later on aspirin, amlodipine, lisinopril, atorvastatin, and sublingual nitroglycerin as needed to follow up in the outpatient clinic.

## Discussion

In a prospective study by Opolski et al., morphological features of 38 MINOCA patients were examined using optical coherence of tomography (OCT) [3]. The mean age of this study was 62 (SD = 13) years, and 55% were female. This is in keeping with our female patient, who falls in the younger spectrum of this age range. This study sought to use OCT, combined with cardiac magnetic resonance (CMR) with late gadolinium enhancement (LGE), to characterize the etiology of MINOCA in this population. They found that the population had a maximal vessel stenosis diameter of 35%, with a small subgroup of this population without any evidence of vessel stenosis [3]. Our patient had no evidence of vessel stenosis on angiography.

They categorized their findings into plaque rupture, which occurred in 24%, and coronary thrombosis, which occurred in about 18%. Furthermore, over 50% of the participants who underwent CMR showed evidence of LGE, a marker of myocardial necrosis or dysfunction [3]. Our patient's electrocardiogram was initially done and was within normal limits. However, a CMR with LGE imaging revealed evidence of acute MI that was isolated to the mid-anterolateral segment, as evidenced by transmural myocardial edema and focal almost-transmural LGE. There were also areas of focal microvascular obstructions within dense necrotic regions of MI with a concomitant regional wall motion abnormality involving the left ventricular segment. The overall ejection fractions were preserved, i.e., LVEF (54%) and RVEF (51%).

In 2019, the American Heart Association (AHA), led by Jacqueline E. Tamis-Holland et al., proposed that the diagnosis of MINOCA should exclude other causes of elevated troponins such as PE, Takotsubo cardiomyopathy, atrial fibrillation, sepsis, myocarditis, or any other overt causes [4]. We ruled out Takotsubo cardiomyopathy with an echocardiogram visualizing normal left systolic function. We obtained a workup for sepsis, including blood cultures, which were all within normal limits. The pro-brain natriuretic peptide (BNP) level was 896 pg/ml, which was not concerning for heart failure. Our patient's lipid profile fits the description of Jacqueline E. Tamis-Holland et al., with total cholesterol of 109 mg/dL (normal < 199 mg/dL) and low-density lipoprotein (LDL) of 42 mg/dL (normal < 99 mg/dL) [4,5].

Outpatient follow-up

Following two months of cardiac rehabilitation, the patient followed up in the outpatient cardiology clinic. She underwent a cardiovascular stress test performed in conjunction with positron emission tomography and myocardial perfusion imaging (PET MPI) with rubidium (Rb82) at rest and post-regadenoson (Lexiscan) infusion. This revealed mild myocardial perfusion abnormality with minimal ischemia in the anterolateral wall. There was an overall preserved coronary flow reserve of 4.20 (coronary flow reserve > 2.0 is considered to be normal), a normal stress ejection fraction (EF) of 73% and resting EF of 71%, and a total coronary calcium score of 0. EKG post-stress test was also negative for ischemia. It is important to note that a non-ST elevation myocardial infarction (NSTEMI) pattern of initial EKG on admission, the use of beta-blockers during follow-up, and a reduced LVEF are useful predictors of long-term outcomes and prognosis [6,7]; our patient fits into the low-risk category.

Guideline recommendation for the management of MINOCA

According to the American College of Cardiology (ACC), there is limited evidence for diagnosis and management due to the paucity of randomized clinical trials involving this subgroup. Typical medications for MI (aspirin, angiotensin-converting enzyme inhibitors, statins, beta-blockers, angiotensin receptor antagonists, and clopidogrel) should be considered based on the etiology of the individual patient [6].

## Conclusions

MI with no obstructing coronary arteries is a clinical presentation that requires individualized management, as etiology varies per patient. Like in our patient, the coronary angiogram was negative for culprit lesions, but a high index of suspicion supported by guideline laboratory and imaging modalities was useful in reaching the diagnosis.
